# The science of childhood and the pedagogy of the state: Postcolonial development in India, 1950s

**DOI:** 10.1111/johs.12246

**Published:** 2019-08-29

**Authors:** Arathi Sriprakash, Peter Sutoris, Kevin Myers

**Affiliations:** 1 Dr Arathi Sriprakash is a Reader in Sociology at the Faculty of Education, University of Cambridge; 2 Peter Sutoris is a Gates Scholar at the Faculty of Education, University of Cambridge; 3 Dr Kevin Myers is a Reader in Social History and Education at the University of Birmingham

## Abstract

This article examines how, in the decade following India’s independence, the psychology of childhood became a locus of experimentation, and an avenue through which approaches to postcolonial development were expressed. Tracing the ideas of educational reformers, psychological researchers and child welfare advocates, we show how a ‘science of childhood’ in this period emphasised both the inherent potential and the emotional complexity of India’s young citizens. However, while identifying this potential, these actors at times circumscribed it by deploying culturalist assumptions about Indian childhood that were linked to a teleology of the new nation state. These were ideas that shaped a ‘pedagogic’ approach to postcolonial modernisation. Nation-building was not just a technocratic undertaking, but an educative project that was scientific, spiritual, and therapeutic in orientation. The article argues for greater attention to the pedagogy of the state in analyses of past and present state-citizen relations.

## INTRODUCTION

In the immediate aftermath of World War II, the newly formed research and education agencies of the United Nations championed a ‘universal’ humanism to repudiate the biological racism that had so violently serviced recent world politics. Through the prism of liberal cosmopolitanism, childhood was imagined as a universal condition, latent with possibilities that were no longer determined by race and that, if nurtured through care and dialogical forms of education, offered a foundation for building peaceful and democratic nations. The coupling of child and society not only reflected the idea of fresh starts popular in post-1945 Europe and in a decolonising world, but it also offered a means for western elites and postcolonial nationalists to express their visions of modernisation and development in pedagogic terms. These terms may have sought distance from colonial paternalism but, in dispensing a kind of therapeutic authority, they proved susceptible to reinscribing racial hierarchies.^1^ In India, the focus of this article, the figure of the child was cast as the figure of the ‘young’ independent state; the child and the state were growing towards a maturity that, with appropriate tutelage, could bring a desired future into being.^2^

The child-state trope has been of some interest to historians and postcolonial theorists. Uday Singh Mehta argues that childhood was a ‘fixed point underlying the various imperial imperatives of education, forms of governance, and alignment with progress’.^3^ Childhood, for liberals in colonial metropoles, connoted an absence of rationality requiring paternalistic guidance. ‘India is a child’, critiques Mehta, ‘for which the empire offers the prospect of legitimate and progressive parenting’.^4^ Thus, he argues, liberalism was able to function in and through imperialism (and patriarchy), providing an ideological defence of colonialism as social progress.^5^ Such stewardship over the development of the child- like colony – and later, nation – was also a stewardship over the future. As Dipesh Chakrabarty has now famously suggested, the historicism in modern European ideas of progress consigns ‘Indians, Africans, and other “rude” nations to an imaginary waiting room of history’- much like a child asked to wait her turn, in this case for political modernity.^6^

If the colony was child-like, then what did this mean for colonised children? Satadru Sen’s analysis suggests that dominant metropolitan assumptions about childhood plasticity and innocence in the mid-nineteenth century were destabilised when taken into colonial India. ‘Native childhood’ was seen as an oxymoron.^7^ Reformatories, boarding schools, and authoritative texts, Sen argues, ‘were paralyzed by an articulation of difference that implied that native children were essentially small, perverse adults’.^8^ Sudipa Topdar’s analysis of late nineteenth century colonial education policy reveals how the Indian child was positioned as potentially ‘seditious’ and thus a source of anxiety for colonial governance.^9^ The decolonisation of Indian childhood, then, required reclaiming the distinction between child and adult that had been obfuscated through colonial hierarchies. India’s child welfare movement taken up by nationalist campaigners could well be re-read as a project oriented toward such reclamation, though, as we discuss, it was not without its own developmental hierarchies and politics of regulation.

As a theory of history, Chakrabarty’s stagist ‘waiting room’ accommodates both the child (as ‘not yet developed’ adults) and colonised states (as ‘not yet developed’ nations) whilst holding western Europe and, increasingly after the Second World War, north America, as the ideal-type. This is a western-centred developmentalism that persists within the normative logics of much present-day research in the field of comparative studies, though not without contestation; reforms of education and child welfare in the postcolonial world continue to be shaped both by tensions with and an incorporation of western universals.^10^ As Ashis Nandy argues, the child in historical and present projects of modern development is simultaneously a projective device and a source of dystopian fear.^11^ The view of the child as ‘reconciling the past and the present of their societies’, we suggest, offers a space to explore what a nation is imagined to be and become, and the pedagogic efforts of states and agencies to ‘tame’ its future.^12^

In this article we focus on how the psychological sciences were enrolled into political efforts to know and govern Indian childhood in the decade following independence in 1947. Childhood was not merely a metaphor for the state, but a site of development practice through which a modern Indian nation – emerging from the violence of colonialism and partition – could be rationally and affectively produced.^13^ Psychology offered a means of relating the individual to the social, the past to the future, and the ‘infinite’ potential of young citizens to the pedagogic – and seemingly therapeutic – role of the state. As K.G. Saiyidain, Joint Secretary and Educational Adviser to the Ministry of Education from 1950 to 1961, reflected,

*‘… the psychological sciences, which reveal to us the complexities of man as a psycho-biological organism and the difficulties inherent in training him, also give us an insight into his infinite possibilities. They underline the fact that he has become the architect of his individual and collective future.’*^14^

Significantly, the analytic universalism of psychology appeared to offer a scientific secularism to frame a new national ‘collective’. . As Saiyidain continued, ‘This truth is significantly recognised both by those who believe in religion and those who reject the idea of any organised God-based religion – by each group in its own way’.^15^

Turning to consider experimental approaches in this period, we look at the Bakubhai Mansukhai (B.M.) Institute of Child Development in Ahmedabad, established by Kamalini Sarabhai, member of a prominent family of industrialists. Sarabhai’s training in psychoanalysis at London’s Tavistock Institute helped to shape the B.M. Institute’s model of child-focused research and practice which aimed to ‘supply a secure foundation for constructive programmes in mental hygiene, education, family and group living’.^16^ The early decades of postcolonial nation-building in India were characterised by widespread educational experimentation in the face of an insufficiently expanded mass schooling system, and the B.M. Institute stands as an example of the institutional and epistemic capacity-building efforts of Indian elites which were distinctly transnational in nature.^17^

Lois Barclay Murphy was an American psychologist who designed and conducted research at the B.M. Institute between 1950 and 55 with an explicit interest in what she called the ‘science of childhood’. Her archive of research notes and papers offers insights into the ways in which connections between child and national development in India were conceptualised by experts in the psychological sciences and brought to their practice. Murphy’s ideas resonated strongly with the growing international political interest in child welfare of the time, linked, as we discuss, to the ascendancy of U.S.-led modernisation which helped the discipline of psychology expand its social authority globally.^18^ Despite Murphy’s self-proclaimed progressive outlook, close friendships with Indian scholars, and dedication to understanding the country, much of her work appeared to reproduce culturalist assumptions and racialised hierarchies, arguably owing to the historicism contained in her comparative method which held the U.S. as the benchmark of modernity.^19^ Of course, theories of modernisation and child psychology were neither read-off as blue-prints for change, nor ‘owned’ by a single constituent. Operating as ‘actors’ categories,’ they offered scope for Indian reformers to shape their own visions for postcolonial development.^20^ Theirs was not a ‘universal’ approach, but a decidedly Indian one, in the context of an elite nationalist project. To demonstrate this, our discussions turn to the work of the Indian Council of Child Welfare to consider how their activities in the 1950s were shaped by postcolonial nationalism, both in terms of an effort to establish Indian leadership on the global stage, and with respect to the educative and therapeutic capacity of the state in reclaiming the specific needs of the Indian child that had been denied through colonial rule. Significantly, these discussions of the ‘pedagogy’ of postcolonial modernisation shine a light on India’s development beyond the more-covered domains of industry and agriculture, to profile the ideas and actions of women reformers whose interest in the most intimate aspects of the life of the new nation are an important but largely missing part of India’s postcolonial historiography.^21^

The paper concludes with a reflection on how ideas of childhood within welfare movements and national development in India in the early postcolonial years have implications for how we understand the ‘pedagogy’ of the state. While much has been written in theoretical terms on state regulation of behaviours, practices and conduct ‘at a distance’,we draw closer attention to the pedagogic orientation of such governmentalities in time and place.^22^ We suggest that elite actors within postcolonial nation-building efforts sought to deploy a therapeutic authority – one that found affinities within strands of educational psychology to engage the unconscious, spiritual and inner worlds of young citizens. This would be a ‘scientific’ approach to national development, but not simply a technocratic, or a narrowly instructional, enterprise.^23^ By unpacking such pedagogic relations of the state, we may come to better know the specific modalities through which an imagined political community *learns* to imagine itself, not least through its children.^24^

### 1.1 Modernisation, psychology, and the development of the child in the 1950s

In this section we examine how the discipline of psychology was enrolled into political ideals about modernisation and linked the domains of child welfare and national development. These were the politics and epistemic frameworks that gave legitimacy to the activities of the B.M. Institute, but they also point to the idea of postcolonial reconstruction as an educative project that was oriented towards both psychological and historical reconciliation.

The normative model of social change expounded by the paradigm of modernisation in the 1950s, especially that emerging from the U.S., was that societies take a linear path of transition from tradition to modernity, and the potential of the individual – particularly their psychological advancement – was as central to this transition as political and economic ‘growth’. As Sara Fieldston explains of this new potentiality:

*‘The collapse of biological theories of human difference gave rise not only to new ideas about child development but also to novel ways of looking at the development of whole populations. No longer were certain groups seen as innately incapable of progress; instead, all peoples of the world, given the proper tutelage, might advance along the path of modernity’*.^25^

What did ‘proper tutelage’ involve? For the U. S, the paradigm of modernisation meant geopolitical programmes of technical assistance which not only delivered large-scale infrastructural projects around the world but also deployed child welfare experts to the decolonising Third World to advise in programmes of research and training.^26^ Occupying the role of educator in matters of child and national development was not, however, a solely American enterprise. As we later discuss, Indian reformers advocated for a specific pedagogy of postcolonial modernisation that would have international reach. And the new international agencies of the UN also saw themselves as having a role in developing expertise in child and community welfare globally.^27^

Indeed, Unesco’s Social Science Department became a major centre of international and comparative research, and its first major undertaking was a research programme to produce, in the wake of the Second World War, an international body of knowledge on the origins of conflict and social tension, and technical planning to prevent it.^28^ Called the ‘Tensions Project’, it drew on the expertise of mainly western social scientists to examine ‘national character’, ‘ways of life’, and the socialisation of communities (with a particular focus on children) across the world. Among the publications associated with the Tensions Project was Margaret Mead’s *Cultural Patterns and Technical Change* which set out to inform technical assistance programmes and development intervention through a ‘conscious application of our new knowledge of human behaviour, derived from the findings of psychiatry, clinical psychology, child development, cultural anthropology and sociology’.^29^ The report contained a section on ‘maternal and child care’ as a key aspect of ‘technical change’, arguing for the ‘urgent need for agencies which will help parents develop new ways of being parents, and children develop new ways of growing up’.^30^ Newly independent India invited and part-financed the Tensions Project to conduct eleven studies led by Indian-based scholars. H.P. Maiti, one of these scholars, would go on to direct the B.M. Institute in Ahmedabad. The studies largely focused on Hindu-Muslim and inter-caste tensions in the aftermath of partition.^31^ American social psychologist Gardner Murphy, husband of Lois Murphy, was appointed in 1950 as a technical consultant on the Indian research undertaken for the Unesco Tensions Project; his role was to serve as expert, critic and teacher in the development of a ‘healthy democracy’.^32^ While the different psychological methods and political implications of the studies were certainly contested by scientists involved in the Tensions Project, Unesco’s activities in this period were notable for their commitment to comparative cross-cultural study, for centring the child as a key agent to bring about desired futures, and for marking out the potential of psychology – particularly American ego-psychology – to facilitate social change globally.^33^

Indian psychologists, who had long been debating the therapeutic methods and cultural theories associated with psychoanalysis in university departments and in forums such as the Indian Psychoanalytical Society, were broadly receptive to the psychological orientation towards child/community/national development contained within the Tensions Project.^34^ The discipline of psychology, with its varied branches, was enjoying a rising profile nationally; the Indian government looked directly to psychological expertise in its social welfare efforts. For example, in January 1951 the Indian government convened a two-day conference, heralded as the ‘first of its kind’ by one participant, which brought together Professors of Psychology to discuss the national coordination of psychological research and teaching in India’s universities.^35^ Proceedings were chaired by Humayun Kabir, poet, philosopher, and Joint Educational Advisor to the Government of India, and 27 delegates from different universities attended in addition to Gardner Murphy. Expanding departments and institutes of Applied Psychology were among the recommendations of the meeting, along with establishing child guidance clinics and a National Psychological Laboratory to coordinate research on ‘national problems’. Education was a key domain of application for psychological concepts and methods. For example, the Central Institute of Education, established in 1947, had quickly developed an active programme of psychological research on topics such as teacher attitudes, student achievement and refugee education.^36^

This was also a period in which state actors and agencies, searching for a means of governing the conduct of its young people against a backdrop of economic and social uncertainty, turned towards the potential of counselling psychology and youth guidance. ‘Student indiscipline’ was a specific area of concern taken up by prominent members of the Ministry of Education such as Humayun Kabir who wrote a series of letters to Chief Ministers of the states from September 1954 to February 1956 with specific suggestions on matters to do with discipline and education. Personal and social discipline had been an ‘obsession’ of nationalist mobilisation; as Ranajit Guha has argued, it was ‘an attempt to compensate by discipline for what the bourgeoisie had failed to gain by persuasion.’^37^ Self-control as a form of spiritual discipline was strategically important to Gandhi’s nationalist campaigns – a ‘weapon for fighting the enemy within’.^38^ Arguably, the rise of psychology enabled the moral prescriptions of elites in post-independence India to take on a more scientific register.

For example, Kabir’s preface in the 1956 publication of his letters on student indiscipline connects individual ‘unity’ with societal ‘health’, presenting a psychologised discourse of education reform:

*In a sense, all education is a process of discipline. The individual is a bundle of instincts, emotions, urges and impulses which have to be coordinated and given a central unity in order to achieve an integrated personality. Society is again composed of individuals and social health depends upon a proper adjustment of the rights and claims of the individuals who constitute it*.^39^

Kabir’s figure of the affective, psychologically complex individual disrupts the assumption of *homo economicus* within normative modernisation frameworks.

In his correspondence, he gives considerable space to the importance of ‘moral and spiritual education’, carefully explained as being part of a secular vision of the state, to ‘inculcate in the young a system of values which binds them together as members of one community’.^40^ He argues for a ‘revival of faith’, because ‘Instruction uninspired by moral and religious values will be inadequate as a preparation for democratic citizenship’.^41^ Kabir ties the morality and spirituality of the individual to the history – and future – of the nation: ‘The reason why India has survived in spite of poverty, hunger, disease and political vicissitudes is her faith in values which transcend the demands of our daily experience’.^42^ These letters on student indiscipline also deal with matters of institutional functioning, such as education management committees, the status of teachers, and the structure of examinations.. Evident within Kabir’s correspondence was the incorporation of spirituality and morality into modern bureaucratic logics of education and state reform; expressions of ‘faith’ that the new nation was attempting to unify – but also discipline – through its institutions.

The bringing together of the material, the psychological and the moral in social reform was also a theme running through the work of Saiyidain who, in 1952, had called for national educational reform that was both ‘technical’ (referring to matters of planning and provisioning) and ‘vital’ (directed towards the psychological life of the nation).^43^

If conventional Freudian psychoanalysis was often understood as a theory of ‘adaptation’, then the work of educational reformers like Saiyidain gave a nod to such science in advocating for psychological – and necessarily historical – reconciliation as a central project of education in the new nation:

*‘The physical, mental and emotional travail through which we have passed during the last few decades, the unsavoury propaganda to which our people were subjected by vested interests, the psychological tensions and mental precipitates left behind by over a century of political subjection and the many unpalatable circumstances attending the birth of our freedom – all these have created a state of cultural confusion and a conflict of values to which no serious minded educationist can remain indifferent’*. *^44^*

Saiyidain’s epistemic resources were not confined to psychoanalysis. Rather, he drew from a wide range of thinkers, such as Felix Adler’s humanistic Judaism, Rabindranath Tagore’s *The Religion of Man*, Henri Bergson’s ideas of creative evolution, and Muhammad Iqbal’s *Secret of the Self* to set out his ideas of ‘vital’ education. Indeed, the science of childhood – its universality but also the pedagogic space it opened up through a recognition of human potential – was here expressed by Saiyidain as a ‘faith and reverence for childhood’.^45^ This was a postcolonial modernisation that did not see spirituality and science as separate.^46^

If politics held science and spirituality together, then it is important to recognise that science drew politics firmly into its domain. We turn now to consider the ‘science of childhood’ at the B.M. Institute of Child Development in Ahmedabad, particularly through the research conducted there by child psychologist Lois Murphy.^47^ Murphy’s notes and reports offer a window into her unshaking belief in science’s ability to describe, examine and ultimately reform childhood. A ‘science of childhood’, Murphy declared, is a social necessity; just ‘as we need a science of engineering in order to make strong enduring bridges, or a science of medicine to cure physical illnesses.’^48^ The self-proclaimed political significance of Murphy’s science was never far from her work; years later she would stress again, ‘We are currently struggling for solution of problems in raising children who can enhance, not destroy, civilization’.^49^

Murphy’s case is significant to consider given she was part of an expansive transnational network of psychologists undertaking research to inform global social reform at the time, and her specific project in Ahmedabad is an example of the epistemic capacity building efforts of the new Indian nation.^50^ Moreover, an analysis of her work offers empirical evidence of Sunil Bhatia’s observations that, while explicit scientific racism may have been dispensed with by post-war psychologists, ‘ethnocentric thinking continued to define the parameters of cross-cultural investigations’.^51^ The historicism and normative developmentalism in Murphy’s work allowed for differentiations in childhood that arguably operated as a new form of cultural racism.

### 1.2 The Indian child and the scientific production of normative development

Lois Murphy’s interest in India predated her interest in psychology. In 1928, she graduated from the progressive and sometimes radical Union Theological Seminary in New York City with a degree focused on Indian religions. It is thus perhaps not surprising that tracing ‘Indianness’ in Indian children was on her mind when she began her research in Ahmedabad. In a draft version of an essay *Child Development in India* later to be published as part of Gardner Murphy’s book for the Unesco Tensions Project, *In The Minds of Men*, a passage that was omitted from the book appeared in which she reflected on her expectations of working in India:

*‘What would I see in children that could connect with the thoughts and feelings of the people of India for the last four thousand years? Was Tagore a dead poet, without spiritual progeny, or influence on children today; would there be some evidence of Gandhi’s hopes for Indian self-sufficiency in the way children are growing up in India now?... In what ways are India’s problems, like western problems, being perpetuated by characteristics rooted in the early development of her people?’*^52^

It was the last question that seems to have largely defined Lois Murphy’s work in India. The idea that a country’s problems could be traced to early-life experiences of its citizens became the driving force behind researching the experience of children growing up in Ahmedabad.

The B.M. Institute—described in Murphy’s reports as pioneering and the only one of its kind in India at the time—focused its research on a series of questions about child development. In the words of H.P. Maiti, the Institute’s director, ‘[o]ur country is passing through the most significant changes in economic and social life. Men are increasingly confronted with new situations and demands, and new values are entering into our mental horizon … What kinds of conflict in value attitudes are likely to emerge in the growing child today? What mental health risks are incidental to the developmental process in the child in our cultural set-up?’^53^ Murphy saw the institute not only asa sign of modernist and scientific progress but also as a continuation of Gandhi’s intellectual and spiritual heritage. ‘I think Gandhiji would approve of its work if he were here,’ she surmised.^54^

Lois Murphy was involved in several studies undertaken at the Institute, but arguably the most significant was the ‘Coping Study.’ Back in the U.S., Murphy worked along with her husband Gardner Murphy at the Menninger Foundation in Topeka, Kansas, where she was the co-director of the Coping Project.^55^ Funded by the National Institute of Mental Health, it was a longitudinal investigation into how ‘normal’ children coped with the stresses of growing up. Research methods utilised in the Indian study included gathering school records and teachers' opinions about academic strengths and weaknesses, classroom observation and observation at play, interviews with family members and school staff, paediatric history, physical examination and observation in a ‘specially designed projective set up.’^56^ Another research project, ‘Study of Customs Relating to the Child,’ focused on the interaction between culture and personality, on the assumption that ‘rapid changes in these customs, as are current at present, are likely to complicate the child’s developmental process and may add to his endopsychic conflict, giving him a confused feeling regarding his status in the society.’^57^ A study entitled ‘Early Childhood Traits as Liked and Disliked by Mother,’ aimed to illuminate ‘what mothers of middle class society in Ahmedabad perceive as good and worthy of approbation, … and as bad and causing worry.’^58^

The location of the B.M. Institute was significant. According to Murphy, Ahmedabad represented a ‘flexibility’ in combining elements of western and eastern ways of life. It was an ‘intensely Indian city, with a long and proud tradition, and on the other hand, a busy laboratory of social change …’^59^ In Murphy’s eyes, the city thus became a site for testing hypotheses about deviations from ‘normalcy’ in childhood. For example, she sought to study ‘the children of working mothers, especially those who spend much of their first two or three years being rocked in a cradle; what does this deprivation of exercise, and chance to get acquainted with the world around them do to the development of their intelligence?’^60^ The findings of experiments conducted here, including the study of the differential impact of growing up in different socioeconomic environments on children, were thought to be relevant well beyond the city, or even India itself.^61^

Murphy’s assessments of the new context and children she saw before her led her to explain ‘difference’ in cultural, and often explicitly hierarchical, terms. For example, in her observations she made many comparisons between Indian and American children. Apart from the many ‘positive’ qualities she noticed in Indian children, including artistic abilities, connectedness to nature and an ability to face challenges without resorting to crying, she identified ‘some striking lacks in the growing up experience of children, as judged from the American point of view. None of the schools I visited gave an opportunity for groups to think together, solving problems, planning work or entertainment … . Group-thinking […] seemed non-existent.’^62^ Furthermore, ‘[w]ithout tools, toys, or any objects of any sort other than kitchen utensils and the few simple implements of agriculture, it is no wonder the children learn little of mechanics of problem-solving in general.’^63^ She was also critical of an ‘impenetrable wall’ of arranged marriages and jobs dictated by caste affiliation that older children and adolescents confronted after their ‘childhood years of freedom.’

The historicism contained within Murphy’s comparative observations was striking in her account of how the breakdown of social structures in post-Independence India created a possible threat to a ‘normal’ childhood. It is worth quoting her at length:

The children-who-used-to-be, the adults of today in India, grew up under a hierarchical authoritative system where the dominance of the British was at least a shadow in the background, and the caste-system and joint family gave the design for living—the pattern and shape of things. The authorities set the limits, established the routines, took care of everyone, settled problems. Without this authority and this pattern, individuals might easily be at sea, rudderless, lost, even disintegrated. And if panicked by failure in satisfyin basic needs to be cared for […] and by the flood of feelings that must have attended the deprivations and struggles of independence and partition, they gave way to primitive violent aggressive feelings and impulses. They did not have the resources of values (civil rights ideals deeply rooted) nor of techniques (group discussion) to deal with these feelings.^64^

In this analysis, British colonialism plays a role only insofar as its decline leads to the breakdown of familiar social realities. Murphy’s argument about the alleged absence of ‘group discussion’ that seems to have been gleaned from anecdotal evidence from Indian public schools that were themselves modelled on British institutions. Her observations ignored or discounted the possibility of group discussion emerging outside of the formal education system, and it is not clear how she arrived at the assertion that Indian society lacked deeply rooted ‘civil rights ideals.’ Nevertheless, Murphy reached a conclusion that under economic and political stress, aggression in Indian adults ‘may burst out in primitive chaotic ways exactly because of the lack of the long slow experiences of patterning that we know’.^65^

Murphy’s research appeared to rely on a model of ‘normalcy’ of childhood which was threatened by social change in the wake of Independence, leaving children unable to ‘cope’ and giving rise to future social tensions. India was not treated merely as a site of tensions that needed to be understood, but also as a testing ground for confirming the universalism of western theories of childhood and pathologising departures from it. The city of Ahmedabad, which Murphy saw as neither modern nor traditional, neither truly westernized nor fully Indian, became a less-developed Other to American research sites. Here, the impact of modernisation and upheaval brought about through decolonisation could be witnessed in real time, forces of history pulling on children from various directions, leaving them supposedly vulnerable to confusion and even prematurely attempting to take control of their circumstances.

This latter point was demonstrated vividly in Murphy’s study on Chandramauli, a seven and a half year old boy who she observed at the B.M. Institute. Chandramauli, she noted, was a ‘bright attractive boy who seems a little girlish in a way’. He struggled with establishing and maintaining relationships with peers, and was ‘inattentive’ and ‘restless’ at school, and ultimately unable to achieve ‘genuine integration and fullfilment’ in both his ‘inner life’ and ‘his dealings with reality.’^66^ A crucial part of Murphy’s analysis of the sources of Chandramauli’s difficulties rested on data derived from an observation session at the Institute during which he was allowed to play without adult interference. Murphy was particularly interested in the effects of Chandramauli’s separation from his father, owing to the latter’s travel to London for postgraduate studies. Murphy identified the boy as ‘repeatedly displaced’, whose experience could stand in for those displaced and separated from family by partition. Strikingly absent in her analysis was a consideration of class-caste background; Chandramauli’s father’s travel to London would have been atypical, yet the ‘universal’ science of childhood did not differentiate. Interpreting how the boy played with toy trains, Murphy seems to suggest that due to the social changes that Independence brought, children felt the need to assume adult roles prematurely; ‘after playing out some problems of change, he announced “I am the driver”, as if in a world that changes, he feels that he must take charge’. These were leadership roles in which children were doomed to fail. In Murphy’s words, they ‘must fail’.^67^

It is difficult to not see Murphy’s conclusions about children as metaphors for the newly created Indian nation- state which British colonisers had predicted was not ready for self-rule. Here, the explicit paternalism of colonialism was replaced by the (largely caste-class blind) therapeutic authority of the modern psychologist. This was a modern expertise that reproduced racial and geopolitical hierarchies whilst being analytically committed to the universal ‘potential’ of the child (and nation). Although much of Murphy’s research from this period appears to have been more diagnostic than prescriptive, her universal ‘science of childhood’ had pedagogic implications for how normative development could be achieved: how India’s citizens could *learn* to live in the best interests of their children and nation. It was precisely the pedagogic possibilities of postcolonial modernisation – and the emerging therapeutic role of the state – that was being debated by Indian reformers in the child welfare movement during these years. However, these positions, as we discuss, stood in some contrast to Murphy’s modernisation thesis by challenging western- centred assumptions of social change.

### 1.3 The welfare of child and nation: Expertise and authority

In this section we look to the activities of the Indian Council for Child Welfare (ICCW) to form a picture of how the figure of the child was linked to therapeutic, spiritual, and nationalist frameworks for social change. The spaces of educational experimentation, child welfare advocacy, and voluntary social work in the years following Independence were profoundly shaped by Indian women from the elite political classes. And yet, arguably because of the dominance of state-centred historiography, the leadership of Indian women in voluntary and quasi-state domains is often overlooked as a central feature of Indian nation-building.^68^ As we discuss, it is within these spaces of expertise and authority that historians may find new ways of understanding how the pedagogic relations between the state and its citizens were conceptualised and contested.

In 1952, the Indian Council for Child Welfare (ICCW) was established as the successor to the 1948 Indian National Committee of the United Nation’s Appeal for Children, building also on the work of the longstanding All India Women’s Conference (AIWC). The first president of the ICCW was Gandhian nationalist and Minister for Health Rajkumari Amrit Kaur, who was joined by Nehru’s daughter and future prime-minister Indira Gandhi as the Vice President, and Hannah Sen, who had been the founding director of Lady Irwin College as General Secretary. Other prominent members of the women-led ICCW included Hansa Mehta, former AIWC president and India’s representative to the 1947–48 UN Commission on Human Rights, and Rameshwari Nehru, Gandhian Nationalist and a founding member of the AIWC.

In December 1952 the ICCW co-convened the International Study Conference on Child Welfare in Bombay with the International Union for Child Welfare under the patronage of the Government of India (which included Rs15,000 of financial support and addresses by Nehru, Saiyidain and other dignitaries).^69^ Attended by some 700 delegates from 26 countries, including representatives from states, non-governmental organisations and international agencies (including the UN and its specialised agencies), the conference was celebrated as the first of its kind to be held in Asia, and was a show of India’s leadership in a global field.^70^ Indeed, it proved to be a significant theatre of Cold War politics; as Oscar R. Ewing, United States Federal Security Administrator, appealed to his audiences in Bombay and back home – care for the child was a ‘force for peace’.^71^ One of the outcomes of the conference was the formation of the Asia Advisory Committee of the International Union for Child Welfare, which had its inaugural meeting in New Delhi in December 1955. It was perhaps the Bandung Conference earlier that year that was on Rajkumari Amrit Kaur’s mind as she opened the meeting with the reflection: ‘As in other fields of activity, it is appropriate that Asian Countries should join hands in the field of child welfare also.’^72^

In the words of Lady Cowasji Jehangir, the 1952 conference aimed to generate ‘better understanding of the all- important problem of child welfare, the pivot on which the national life and progress of the country depends’.^73^ Thematic sessions over the seven days included discussions on health and social services to meet basic needs, the care and education of the ‘handicapped’ child, and parental education and child development in the home, with speakers typically showcasing the activities and progress made by the agencies they represented. Many asserted the importance of non-governmental, voluntary efforts in the domain of child welfare, and while the scientific frameworks for approaching child welfare were as diverse as the congregation, participants repeatedly underscored the social and political significance of securing the welfare of the child.^74^ It was thus a forum at which epistemic authority over matters of child welfare was being negotiated alongside the political authority to guide social change nationally and globally.Indeed, the conference revealed uncertainties among some Indian reformers about the role of experts in matters of child welfare. Saiyidain, in his plenary address, challenged the authority of expertise that was otherwise being celebrated at the gathering: ‘Expert knowledge can certainly build a bridge or an atom bomb, but not a human personality for which love alone can provide the necessary insight’.^75^ This may well have been a caution against an excessive instrumentalism of ‘science’ that privileged the ‘technical’ over the ‘vital’ within his schema of education reform, but it could have also been an expression of unease about the state and its diverse (and to some extent self-styled) experts exerting unreflexive authority over the intimate worlds of citizens. Saiyidain also seemed to warn against culturalist modes of understanding ‘difference’ in childhoods, reiterating a point made by Nehru in his opening address days before: that national reconstruction and the welfare of all children requires addressing widespread poverty; ‘revolutionising the entire economic structure’.

In the face of material inequality, it was recognised that the state had to carefully consider its approach towards institutionalising practices of care. ‘The best place for the child to grow up is his natural home, under the care of his own parents’, reaffirmed Rajkumari Amrit Kaur.^76^ Public education, rather than direct intervention, were foregrounded within the activities of the ICCW. In 1954, it established a Child Bureau in Delhi to facilitate national and international knowledge exchange on child welfare (assisted by a Rs15,000 grant from the Central Social Welfare Board) and appointed a Field Consultant for India and Other Asian countries to coordinate regional activities ‘systematically and scientifically’.^77^ It also produced public educational materials in the form of editorials and monthly newsletters, and television and radio messages for National Children’s Day activities, and proposed the publication of pamphlets in Hindi (for ‘the parents of lower middle-class groups’) on child care – covering topics such as diet, hygiene, exercise and ‘emotional security and affectionate understanding’.^78^ ‘Raising the standard of parental care’ was discussed as a ‘vital’ subject of research and action at the 1955 Asia Advisory Committee meeting.

Amrit Kaur, however, cautioned that ‘existing patterns of family life may be disturbed if too much outside guidance and assistance are provided for the parent’. Central to India’s socialism of the time was the idea that voluntary effort and self-help were crucial to nation-building.^79^ Here, Amrit Kaur explicitly linked the pedagogic efforts of child welfare to ‘the principle of self-help’. She was conscious that programmes of national reconstruction designed by national and international elites should not take on a paternalistic authority associated with colonial rule but instead encourage India’s new citizens to embody their independence: ‘the tendency to depend unduly on government has often impeded the growth of independent and voluntary co-operative action’.^80^ The pedagogic role of the state was to help people help themselves. As Nikhil Menon has argued, the entwined discourse of national planning and self-help was, for the Indian state, ‘a means of instructing its citizenry on the correct terms of civic participation’.^81^

However, this was not an approach to social reform that was narrowly conceptualised in behaviourist, instructional terms. Our analysis of child development discourses illustrates that there were specific pedagogic modalities brought to the state-citizen relationship in the context of Indian postcolonial development; namely, modalities that sought to engage, in different ways, with the inner-self. As we turn now to reflect on these approaches, we suggestthat a more nuanced understanding of the ‘pedagogy’ of the state can be useful to historical sociologists seeking to examine such practices – and contingencies – of state-making.

### 1.4 Pedagogies of the postcolonial state: Scientific, spiritual, therapeutic

While much has been written in the fields of historical sociology and political science about the shifting relations between the citizen and state, there has been less attention paid specifically to the role of education as an institution through which this relation is mediated, and less still to the ways in which pedagogic relations extend into social life. Jessica Pykett offers a rare analysis of the ‘pedagogical state’, drawing our attention to how ‘teacherly’ functions of the state – the pedagogical forms of power – are mobilised to govern citizens' conduct and produce emerging forms of citizenship. She argues,

*Because pedagogy cannot be reduced to teaching, learning or education, it provokes us to consider not simply the disciplining and directive facets of education, but also the way pedagogy is used in order to develop competences and capabilities and to empower subjects in their future self-directed knowledge, experience and activities*.^82^

Pykett’s account usefully draws attention to how a pedagogic relation might seek to establish authority, but can, albeit conditionally, involve critique and change. This is the ever present ‘shine and shadow’ of educational relationships that can helpfully avoid reading-off governmentality as a totalising force.^83^ Bringing these ideas to our analysis, we identify at least three pedagogic orientations of the postcolonial Indian state in the 1950s within the discourses of educational reformers, child development researchers, and child welfare advocates.

Firstly, assisted by the new psychological sciences of childhood, the universal *potential* of citizens – young and old – was identified and underscored by educational reformers. This enabled the postcolonial state to manoeuvre away from the explicit paternalism of colonial authority but maintain the educative capacity of its elite representatives and legitimise their expertise. Of course, its pedagogic approach, which was ‘progressive’ in so far as it recognised the capacity of its citizens, was not necessarily liberatory or transformative. In fact, the assumed universality of the psychological sciences and an emphasis on the inner self enabled education reformers and researchers to skirt around the lived realities of structural inequality and material deprivation. Instead, structural inequality came to be seen as itself constitutive of the psychology of the nation, as we saw in Saiyidain’s concerns over the ‘cultural confusion’ of the Indian population and in Murphy’s accounts of its ‘primitive’ impulses. Arguably, this enabled Indian and international elites to assume the role of the steady educator of the subaltern masses who were presumed to be in need of guidance, thereby reinstalling a pedagogic paternalism.

Secondly, this was a pedagogic relationship that was required to incorporate the diversity of its learner-citizens. Spirituality was not merely ‘added to’ normative frameworks of modernisation, but rather, postcolonial modernisation was itself understood through the spiritual life of the self and the nation. In other words, key to the demos recognising itself in the narrative of the new democracy was the incorporation of its spiritual pluralism within the framework of the modern nation-state. Scientific secularism (offered by the universal science of childhood) and economic planning (as the official mode of centralised reform) would have arguably been an ineffective mode of governance without a pedagogy of the state that recognised – in rhetoric if not in practice – both the spiritual pluralism and the universal capacity of the people it called upon.

Thirdly, the pedagogy of postcolonial modernisation appeared to be self-consciously therapeutic; it involved coming to terms with the past in order to embody independence for the future. This not only resonated with the nationalist principles of self-help being advocated for in a context of material scarcity, but it was also an investment in the idea of self-potential and emotional integration that was being reaffirmed by the psychological sciences of the time. Inner adaptation was given space alongside behavioural change in developmentalist discourses of the planning state. The approach, then, of postcolonial nation-building was neither purely technocratic, nor narrowly instructional. It sought to address, rather than eschew, the spiritual, unconscious, and inner dynamics of social change.

There is of course more to know about the educative project of national development; more to fill in of the capacities and limitations of the scientific, spiritual and therapeutic pedagogy of the postcolonial Indian state. To be sure, analyses of how actors – elite and subaltern – conceptualise and relay the state pedagogically also need to be alive to how the state is contested and learned, to the constitutive tensions of force and consent.^84^ We argue that training our attention to the pedagogic relations of the state means we cannot foreclose the capacities of actors to both impart and acquire, and thereby to constantly remake, their imagined political communities.

## ENDNOTES

1 Following Miller and Rose, therapeutic authority refers to forms of governance – or ‘acting upon the actions of others’– in which an individual’s relations with others is taken to ‘intersect with their relations with themselves’ (p.29). The connection between the self and the nation, as this paper demonstrates, was a recurrent preoccupation of Indian reformers. Miller, P. and Rose, N. ‘On therapeutic authority: psychoanalytical expertise under advanced liberalism’. *History of the Human Sciences*, vol. 7, no. 3, 1994, pp. 29–64. An historical case study of the therapeutic authority within state-citizen relations is offered by Michal Shapira’s analysis of British postwar reconstruction in *The War Inside: Psychoanalysis, Total War, and the Making of the Democratic Self in Postwar Britain*. (Cambridge: Cambridge University Press, 2013).

2 See, for example, Srirupa Roy’s characterisation of the ‘infantile citizen’ in Nehruvian India in *Beyond Belief: India and the Politics of Postcolonial Nationalism*. (Durham: Duke University Press, 2007), p.22. An overview of infantilising tropes in colonial and neo-colonial discourse is offered by Matt Finn and Cheryl McEwan, ‘Left in the Waiting Room of History? Provincializing the European Child’, *Interventions: International Journal of Postcolonial Studies*, vol. 17, no. 1, 2015, pp.113–134. Peter Sutoris has also written on state-sponsored documentary film as a form of public education in the early postcolonial years in *Visions of Development: Films Division of India and the Imagination of Progress, 1948–75*. (New York: Oxford University Press, 2016).

3 Uday Singh Mehta, *Liberalism and Empire: A Study in Nineteenth-Century British Liberal Thought*. (Chicago: University of Chicago Press, 1999), p. 31.

4 *Ibid*. p. 32.

5 See also David Theo Goldberg, ‘Liberalism’s limits: Carlyle and Mill on “the negro question”’, *Nineteenth Century Contexts*, 22:2, 2000, pp. 203–216.

6 Dipesh Chakrabarty, *Provincializing Europe: Postcolonial Thought and Historical Difference*. (Princeton: Princeton University Press, 2000), p. 8.

7 Satadru Sen, *Colonial Childhoods: the Juvenile Periphery of India* 1850–1945, (London: Anthem Press, 2005), p. 1.

8 *Ibid*, p.1

9 Topdar’s analysis also offers insight into the pedagogic efforts of the colonial state: ‘Textbooks of ‘aggressive character’ were censored and substituted by those imparting lessons on ‘state feeling’ and duties of a ‘good citizen”. Sudipa Topdar, Duties of a ‘good citizen’: colonial secondary schooltextbook policies in late nineteenth-century India, South Asian History and Culture, 6:3, 2015, 417–439. For other studies on childhood in India, see the collection ‘Childhoods in India: Contemporary and Historical Perspectives’ in *South Asian History and Culture* 6:3, 2015.

10 See critiques of developmentalism in comparative education in: Erica Burman, *Developments: child, image, nation*. (Hove: Routledge, 2008); and, Takayama, K., Sriprakash, A., and Connell, R.W., ‘Towards a Postcolonial Comparative Education’, *Comparative Education Review*, vol. 61, no. S1, 2017.

11 Ashis Nandy, ‘Reconstructing Childhood: a critique of the ideology of adulthood’. *Alternatives X*. Winter, 1984, pp. 359–375.

12 *Ibid*. p. 366. Beyond the Indian context, Joanne Faulkner’s analysis of the centrality of childhood to the project of settler- colonialism in Australia reveals how a ‘heightened anxiety’ about childhood is ‘*integral* to anxiety about white possession of Australia’. See *Young and Free: (post)colonial ontologies of childhood, memory, and history in Australia*. (London: Rowman &Littlefield, 2016)

13 The making of the postcolonial state involved the ‘distribution of sentiments and desires’ as part of modern scientific reason. See Stoler, A.L. ‘Affective States’, p.9, in *A Companion to the Anthropology of Politics*. (eds.) Nugent, D. and Vincent, J. (Oxford: Blackwell Publishing, 2007), pp. 4–20. On science and planning in India, see Gyan Prakash *Another Reason: Science and the imagination of modern India*, (Princeton: Princeton University Press, 1999).

14 Saiyidain, K.G. *The Man in the New World*, (Bombay: Asian Publishing House, 1964), p.20.

15 *Ibid*. p. 20.

16 Quarterman, K.D. and Wright, J.W. ‘B.M. Institute of Child Development’*. Indian Journal of Pediatrics* vol. 23, 1956, p. 150.

17 In 1951 the national Gross Enrolment Ratio for Elementary Schooling (Standards I – VIII) was 32% and 17.7% for females. The national literacy rate in 1951 was 18.3% (and just 8.9% for women). Figures drawn from *Educational Statistics at a Glance*, (Government of India, Ministry of Human Resource Development). Available at http://mhrd.gov.in/statist (accessed 26 November 2018). SeeTaylor Sherman’s discussion of educational experimentation and expansion in ‘Education in early post-colonial India: expansion, experimentation and planned self-help’, *History of Education*, vol. 47, no. 4, 2018, pp. 504–520. Rosie Peppin Vaughan has written of the transnational collaborations of education reform in ‘Complex Collaborations: India and International Agendas on Girls’ and Women’s Education, 1947–1990‵, *International Journal of Educational Development* vol. 33, 2013, pp.118–29. See also Damiano Matasci and Joëlle Droux’s analysis of the transnational networks surrounding childhood and education in ‘(De)Constructing the global community: Education, Childhood and the Transnational History of International Organizations' in: Eckhardt Fuchs and Eugenia Roldán Vera (Eds.) *The Transnational in the History of Education: concepts and perspectives*. (Netherlands: Palgrave Macmillan), 2019, pp.231–260.

18 Ellen Herman, E. *The Romance of American Psychology. Political Culture in the Age of Experts*. (Berkeley: University of California Press, 1995). p. 251.

19 Such historicism is identified by Sunil Bhatia as Orientalism that persists in Euro-American psychology and has been internalised by psychologists in the non-west. See ‘Orientalism in Euro-American and Indian Psychology: historical representations of ‘natives' in colonial and postcolonial contexts. *History of Psychology*. 5:4, 2002, p.376–398.

20 Here we borrow Duncan Bell’s useful framing of political theory as an ‘actor’s category’ to emphasise how it defies reductive singularity and is instead filled with meaning by people in place and time. See Duncan Bell, *Reordering the World: Essays on Liberalism and Empire*, (Princeton, Princeton University Press, 2016).

21 However, an important contribution, and particularly relevant to this paper, is Kim Berry’s analysis of the translations of US-based programmes of ‘home science’ in India in the 1950s as part of transnational development aid that saw not only the production of the ‘ideal woman leader’ but also an Indian vision of modernity and nation-building. See: ‘Lakshmi and the Scientific Housewife: a transnational account of Indian women’s development and production of an Indian modernity’, *Economic and Political Weekly* Vol. 38, No. 11 (Mar. 15–21, 2003), pp. 1055–1068.

22 Nikolas Rose and Peter Miller. ‘Political Power beyond the State: Problematics of Government.’ *The British Journal of Sociology* vol. 43, no. 2, 1992, pp. 173–205.

23 For a discussion of how citizens were called into the centralised economic planning of Nehruvian developmentalism, see Nikhil Menon, ‘Help the Plan – Help Yourself’: Making Indians Plan Conscious', in *The Postcolonial moment in South and Southeast Asia*, (eds.) Prakash, G., Menon, N., and Laffan, M. (London: Bloomsbury Press, 2018), pp. 221–242.

24 We refer here to Benedict Anderson’s well-known *Imagined Communities: Reflections on the Origin and Spread of Nationalism* (London: Verso, 1983).

25 Sara Fieldston, *Raising the World: Child Welfare in the American Century*. (Cambridge: Harvard University Press, 2015), p. 143.

26 *Ibid*.

27 Erik Linstrum reports that the UN technical assistance budget in 1949–1950 allocated US$1.6 million for the training of psychologists, psychiatrists, and psychiatric social workers. See *Ruling Minds: Psychology in the British Empire*, (Cambridge: Harvard University Press, 2016), p. 203.

28 Teresa Tomas Rangil, ‘The Politics of Neutrality: UNESCO’s Social Science Department, 1946–1956’, *CHOPE Working Paper* No. 2011–08, April 2011. Available at: https://hope.econ.duke.edu/node/132 (Accessed 26 November 2018); see also Perrin Selcer, ‘The View from Everywhere: Disciplining Diversity in Post World-War II International Social Science’ *Journal of the History of the Behavioural Sciences*, vol. 45, no. 4, 2009, pp. 309–329.

29 Margaret Mead, *Cultural Patterns and Technical Change: a manual prepared by the World Federation for Mental Health*, (Paris: Unesco, 1953), p.8.

30 *Ibid*. p. 302.

31 ‘Indian National Commission for Unesco. Project for Research on Social Tensions’. Unesco/SS/4 Paris, 13 November, 1951.

32 Unesco Archives, Paris, 327.5: 301.18 A 53, Tensions affecting Int. Understanding Community Studies Part II - from 1/XI/49; Angell to Pendleton Herring 17 Nov 1949.

33 See Myers, K., Sriprakash, A., Sutoris, P. (forthcoming) ‘Towards a new humanism: time and emotion in decolonising India, 1947–1951’ for a more detailed analysis of Unesco’s research activities in India.

34 For historical analyses of psychoanalytic ideas in India, see: Christiane Hartnack, ‘Vishnu on Freud’s Desk: Psychoanalysis in Colonial India’, *Social Research*, vol. 57, no. 4, 1990, pp. 921–949; and Shruti Kapila, ‘The Enchantment of Science in India’, *Isis*, no. 101, 2010, pp. 120–132.

35 National Archives of India (hereafter, NAI), D.842/51-GIII. Comments made by Dr Radhanath Rath, Ravenshaw College, Cuttack. *Draft Report of the Psychology Conference – Comments of the members*. While a full list of delegates were not found in the available archival material, comments were received by ten members, all male, including three who were undertaking research for the Unesco Tensions Project (Pars Ram, H.P. Maiti, and Kali Prasad).

36 *Studies in Education and Psychology. Summary of Reports*. (Delhi: Central Institute of Education, 1952).

37 Ranajit Guha, *Dominance without Hegemony: history and power in Colonial India*. (Cambridge: Harvard University Press, 1997), p. 135.

38 *Ibid*. p. 136.

39 Humayun Kabir *Letters on Discipline*. (Delhi: Ministry of Education, Government of India and Albion Press, 1956), p. ii. See also Kabir’s *Indian Philosophy of Education* (New York: Asia Publishing House, 1961), a collection of essays and speeches in which he elaborates his positions on national unity with respect to education.

40 *Ibid*. p. 29. Kabir’s explanation of moral and spiritual education being part of a secular state is in somewhat pragmatic terms: ‘Religion is part of life and it is but natural that children will ask questions about it’ (p.30). He also positions moral and spiritual education as a unifying force: ‘the emphasis on the secular nature of the State demands a recognition of the spiritual values which are common to all faiths’.

41 Kabir’s provisional list of the values underpinning ‘moral and spiritual education’ that are needed for the ‘modern world’ include: ‘respect for human personality, faith in moral responsibility, respect for common consent, devotion to truth, respect for excellence, moral equality, pursuit of the general welfare and spiritual enrichment’. *Ibid*. p.31.

42 *Ibid*. p.29

43 K.G. Saiyidain, *Education, Culture and the Social Order*. (Bombay: Asia Publishing House, 1952).

44 *Ibid*. p. xv.

45 *Ibid*. p. 201.

46 For a discussion of the spiritual dimensions contained within socialist thought in postcolonial India, see Taylor Sherman, ‘”A new type of revolution”: socialist thought in India, 1940s – 1960s’, *Postcolonial Studies*, 2018, DOI: 10.1080/13688790.2018.1500085

47 As a consequence of the material available in the archives consulted, our discussions here focus on Murphy’s interpretations and practice of the science of childhood rather than those of the Indian researchers and practitioners who were also working at the Institute in the 1950s. The resulting analysis continues to be structured, therefore, by the politics of the colonial archive. See, Laura Ann Stoler ‘Colonial Archives and the Arts of Governance.’ *Archival Science* vol. 2 nos. 1–2, 2002, pp. 87–109.

48 Kansas Historical Society Archives (hereafter KHS Archive), Gardner Murphy Papers, 118–4–5-6 India Reports (1955), Needs of Children in a Changing City, p. 4.

49 Lois Barclay Murphy, ‘Some mutual contributions of psychoanalysis and child development.’ *Psychoanalysis & Contemporary Science*, no. 2, 1973, pp. 99–123.

50 See Myers, K., Sriprakash, A., Sutoris, P. (forthcoming) for an account of these transnational networks facilitated by Unesco.

51 Bhatia, 2002, p.387.

52 KHS Archive, Gardner Murphy Papers, 118–4–5-6 Child Development in India, p. 1–2. Gardner Murphy’s book was published as *In the Minds of Men: The Study of Human Behaviour and Social Tensions in India*, (New York: Basic Books, 1953).

53 KHS Archive, Gardner Murphy Papers, 118–4–5-6 India Reports (1955), BM Institute of Child Development, Review of Current Research Projects [hereafter ‘Review of Current Research Projects’], H.P. Maiti, p. 1.

54 KHS Archive, Gardner Murphy Papers, 118–4–5-6 India Reports (1955), Needs of Children in a Changing City, p. 4.

55 Coping was defined in this study as the ‘way in which the child orchestrates or integrates direct and indirect efforts and defense mechanisms in dealing with external pressures and internal conflicts or stress’ (Review of Current Research Projects, p. 2).

56 Review of Current Research Projects, p. 3.

57 Review of Current Research projects, p. 6.

58 Review of Current Research Projects, p. 8.

59 KHS Archive, Gardner Murphy Papers, 118–4–5-6 India Reports (1955), Ahmedabad Today, p. 2.

60 KHS Archive, Gardner Murphy Papers, 118–4–5-6 India Reports (1955), Needs of Children in a Changing City, p. 2.

61 *Ibid*.

62 KHS Archive, Gardner Murphy Papers, 118–4–5-6 Child Development in India, p. 10.

63 KHS Archive, Gardner Murphy Papers, 118–4–5-6 Child Development in India, p. 11–12.

64 KHS Archive, Gardner Murphy Papers, 118–4–5-6 Child Development in India, p. 14.

65 *Ibid*.p. 8.

66 KHS Archive, Gardner Murphy Papers, 118–4–5-6 Chandramauli, p. 1–4.

67 *Ibid*.

68 Taylor Sherman, ‘Not Part of the Plan? Welfarism, Women, and Indian Socialism in the Nehru Years’, forthcoming.

69 Mithan J Lam, Jt Hon Secretary International Study Conference on Child Welfare Bombay. Letter to the Editor: Child Welfare *The Times of India;* Sep 22, 1952; pg. 4.

70 Countries that delegates came from included: Australia, British West Indies, Burma, Egypt, Indonesia, Iran, Iraq, Japan, Malaya, Netherlands, Pakistan, Portugal, Sweden, Sudan, Thailand, the U.S., and Yugoslavia. Delegates from Ceylon and the Philippines were registered but were unable to attend.

71 A force for peace seen in child care: Ewing tells India conference not to allow economic gains to curb human relations. *New York Times;* Dec 12, 1952; pg. 39. See also Fieldston, *Raising the World*, 2015.

72 Nehru Memorial Museum and Library Archives (hereafter NMML). All India Women’s Conference (IV Inst.) Subject File, 214. Papers relating to Advisory Committee on Child Welfare in Asian countries. Rajkumari Amrit Kaur. Inaugural address to the Asia Advisory Committee of the International Union for Child Welfare – meeting in New Delhi 15–19 December 1955.

73 *International Study Conference on Child Welfare Report of the Proceedings*, Bombay 5–12 December 1952. (Geneva: International Union for Child Welfare, 1952).

74 The first resolution of the Conference, in reaffirming the Declaration of the Rights to the Child, was to emphasise and maintain the work of voluntary agencies to ‘supplement governmental effort’ in child welfare. *Ibid*. p.222

75 *Ibid*. p.145.

76 NMML. All India Women’s Conference (IV Inst.) Subject File, 214. Papers relating to Advisory Committee on Child Welfare in Asian countries. Rajkumari Amrit Kaur. Inaugural address to the Asia Advisory Committee of the International Union for Child Welfare – meeting in New Delhi 15–19 December 1955.

77 *Ibid*.

78 NMML. All India Women’s Conference (IV Inst.) Subject File, 214. India Council for Child Welfare. The Plan of Children’s Bureau, 1954.

79 Taylor Sherman, ‘Education in early postcolonial India’, 2018.

80 NMML. All India Women’s Conference (IV Inst.) Subject File, 214. Papers relating to Advisory Committee on Child Welfare in Asian countries. Rajkumari Amrit Kaur. Inaugural address to the Asia Advisory Committee of the International Union for Child Welfare – meeting in New Delhi 15–19 December 1955.

81 Nikhil Menon, ‘Help the Plan – Help Yourself’, 2018, p. 222.

82 Jessica Pykett. 2010. Introduction: the pedagogical state: education, citizenship, governing. *Citizenship Studies*. 14:6, 617– 619. See also: Jessica Pykett. 2010. Citizenship Education and narratives of pedagogy, *Citizenship Studies*, 14:6, 621–635

83 The idea of the ‘shine and shadow’ draws on Indigenous scholarship that has demonstrated that the ‘shine’ of modernity cannot be delinked from its ‘shadow’ of ‘expansionist control of lands, racism and epistemic violence’, as the analysis by Ahenakew, C., V. Andreotti, G. Cooper, and H. Hireme powerfully demonstrates in ‘Beyond Epistemic Provincialism: De- Provincializing Indigenous Resistance’. *Alter Native* 10 (3): 216–232, 2014. The ‘shine and shadow’ of educational relationships is explored in Rudolph et al. ‘Knowledge and racial violence: the shine and shadow of ‘powerful knowledge”, *Ethics and Education*, 2018, Online First DOI: 10.1080/17449642.2018.1428719.

84 Ranajit Guha, *Dominance without Hegemony*, 1997, p.103

